# A cross-cultural analysis of the relationship between the level of depression and attitudes toward death in cancer patients

**DOI:** 10.1192/j.eurpsy.2024.1343

**Published:** 2024-08-27

**Authors:** K. Kholmuradova

**Affiliations:** Psychology, Moscow State University, Tashkent, Uzbekistan

## Abstract

**Introduction:**

♦Over the last 10 years, the number of cancer patients in the world has increased by almost 23%, and the number of cancer deaths has also increased by about 10%. Malignant neoplasms still remain as one of the main causes of mortality in the population. Patients with oncopathology are characterized by a high level of depression which leads to inadequate attitudes towards the disease and its treatment, and this may further act as a risk factor for disease susceptibility and aggravate its course (Schulz-Kindermann, 2021). It is relevant to search for variables that act as a personal resource in coping with cancer. It is hypothesized that one such personal resource is the specificity of attitudes towards death.

**Objectives:**

To conduct a comparative analysis of the relationship between the level of depression and the peculiarities of the attitude to death in cancer patients in Russia and Germany.

**Methods:**

- Beck Depression Inventory to determine the level of depression severity.
- Death Attitude Profile-Revised to determine the type of attitude to death.

For statistical processing of data, the SPSS 23.0 statistical package was used with a preliminary check for normality of distribution using the Kolmogorov-Smirnov statistical criterion.

**SELECTION:**

The sample consisted of a total number of 50 cancer patients with 25 each undergoing treatment in Russia (Moscow) and in Germany (Munich). The study was based on the sample obtained from the P. A. Herzen Moscow Research Oncological Institute and the Helios Munich-West Clinic. Overall, the sample was relatively gender-balanced.

**Results:**

The following results were obtained from the study:

1. The mean value of depression level in cancer patients is higher in Russia than in Germany.
2. The level of depression in cancer patients in both the countries is correlated with:
  - marital status (p=0.36)
  - stage of disease (p=0.001)
  - type of treatment (p=0.001)
  - belief in God (p=0.024)
  - adherence to a particular religious denomination (p=0.008)
3. The level of depression was correlated with a certain type of attitude towards death: a higher level of depression was associated with scores on the “fear of death” scale (p=0.000), and a lower level (or lack of ) with the “neutral acceptance of death” scale (p=0.000)
4. The fear of death is seen to be most common in the sample of patients from Russia, while the neutral acceptance of death is more prevalent in the sample from Germany.

**Conclusions:**

The results suggest that a positive attitude to death (neutral as one of these types) is correlated, along with other factors, with lower levels of depression, which may be a personal resource in coping with the disease.

This allows us to make the assumption that when providing psychological support to cancer patients, it is necessary to pay attention not only to the attitude to life and illness, but also to the attitude to death.

**Disclosure of Interest:**

None Declared

